# Recent Technical Advances in Accelerating the Clinical Translation of Small Animal Brain Imaging: Hybrid Imaging, Deep Learning, and Transcriptomics

**DOI:** 10.3389/fmed.2022.771982

**Published:** 2022-03-24

**Authors:** Wuwei Ren, Bin Ji, Yihui Guan, Lei Cao, Ruiqing Ni

**Affiliations:** ^1^School of Information Science and Technology, ShanghaiTech University, Shanghai, China; ^2^Shanghai Engineering Research Center of Energy Efficient and Custom AI IC, Shanghai, China; ^3^Department of Radiopharmacy and Molecular Imaging, School of Pharmacy, Fudan University, Shanghai, China; ^4^PET Center, Huashan Hospital, Fudan University, Shanghai, China; ^5^Shanghai Changes Tech, Ltd., Shanghai, China; ^6^Institute for Regenerative Medicine, University of Zurich, Zurich, Switzerland; ^7^Institute for Biomedical Engineering, ETH Zürich and University of Zurich, Zurich, Switzerland

**Keywords:** deep learning, magnetic resonance imaging, multimodal imaging, neuroimaging, positron emission tomography, optoacoustic imaging, image registration, fluorescence imaging

## Abstract

Small animal models play a fundamental role in brain research by deepening the understanding of the physiological functions and mechanisms underlying brain disorders and are thus essential in the development of therapeutic and diagnostic imaging tracers targeting the central nervous system. Advances in structural, functional, and molecular imaging using MRI, PET, fluorescence imaging, and optoacoustic imaging have enabled the interrogation of the rodent brain across a large temporal and spatial resolution scale in a non-invasively manner. However, there are still several major gaps in translating from preclinical brain imaging to the clinical setting. The hindering factors include the following: (1) intrinsic differences between biological species regarding brain size, cell type, protein expression level, and metabolism level and (2) imaging technical barriers regarding the interpretation of image contrast and limited spatiotemporal resolution. To mitigate these factors, single-cell transcriptomics and measures to identify the cellular source of PET tracers have been developed. Meanwhile, hybrid imaging techniques that provide highly complementary anatomical and molecular information are emerging. Furthermore, deep learning-based image analysis has been developed to enhance the quantification and optimization of the imaging protocol. In this mini-review, we summarize the recent developments in small animal neuroimaging toward improved translational power, with a focus on technical improvement including hybrid imaging, data processing, transcriptomics, awake animal imaging, and on-chip pharmacokinetics. We also discuss outstanding challenges in standardization and considerations toward increasing translational power and propose future outlooks.

## Introduction

Clinically deployed imaging modalities, including MRI, PET, and single-photon emission CT (SPECT), have vastly facilitated the understanding of human brain function (1–3) and the development of disease biomarkers for brain disorders such as brain tumors, Alzheimer’s disease, ischemic stroke, and multiple sclerosis toward personalized medicine **(4)**. MRI sequences for brain imaging, such as diffusion-weighted imaging (DWI), diffusion tensor imaging (DTI) for white matter integrity (5), structural T_1_ and T_2_ for regional brain atrophy, arterial spin labeling (ASL) for cerebral perfusion, and susceptibility-weighted imaging for microbleed assessment, are routinely performed both in animal models in the laboratory and in human patients in the clinical setting (6). The development of 7 T human MRI allows imaging of the living human brain at the mesoscopic level with a high spatial and temporal signal-to-noise ratio (SNR) (7, 8). There are currently seven Food and Drug Administration approved commercially available MR contrast agents with indications for central nervous system lesions (9). This gadolinium (III)-based contrast agents are widely used in the clinical setting. In addition, targeted agents, activatable agents, high-relaxivity agents, and gadolinium-free MR contrast agents are being developed (9). Gadolinium(III)-based contrast agents have been successful as they provide essential diagnostic information that often cannot be obtained with other non-invasive techniques. PET has been widely used as a highly quantitative non-invasive tool to detect neurotransmitter receptors and protein/enzyme levels, e.g., cerebral glucose metabolism ([^18^F]fluorodeoxyglucose, FDG), dopamine receptor ([^11^C]raclopride), glial activation, and amyloid-β plaques, in the living human brain (10). It is also noted that optical imaging accounts for a large segment of clinical imaging and has been used in image-guided surgery, although far fewer contrast agents have been approved for optical imaging than PET (11, 12). Emerging optical imaging methods, including fluorescence and optoacoustic imaging (OAT) (13, 14), have shown increasing value for assisting diagnosis and surgical navigation. Observation of molecular, structural, and functional changes in the brains of small animal models that recapitulate human diseases is highly valuable for understanding physiological function (15) and the mechanisms underlying brain disorders (16). Small animal brain imaging has been indispensable for the development of novel therapeutic drugs and diagnostic imaging probes. A previous study showed that preclinical dosimetry studies and models facilitate the prediction of clinical doses of new PET tracers (17). Here, we summarize the gaps in the translation of preclinical neuroimaging and focus on the recent technical developments in improving its clinical relevance, especially regarding data acquisition (hybrid imaging and awake animal imaging), data analysis using deep learning (DL), and transcriptomics. We also outline the current outstanding challenges in closing the translational gap and propose outlooks for the future.

## Hybrid Imaging

The most commonly used hybrid systems in small animal imaging are the integration of molecular imaging using PET, SPECT, fluorescence molecular tomography (FMT), and OAT with structural imaging using MRI and CT. Both PET/SPECT-CT and PET/SPECT-MRI provide research tools for probing molecular and structural information and have demonstrated significant value in brain research (18, 19). Molecular imaging modalities generally lack high-spatial resolution and soft tissue contrast to accurately allocate the distribution of specified molecular signals. Thus, it is essential to provide accurate anatomical information along with the molecular imaging modalities to better interpret the acquired molecular signals (20–22). There are two approaches to address this: sequential-mode using different standalone modalities and follow-up image processing/image registration (23) or hybridized multimodal imaging (24). Sequential-mode multimodal imaging allows convenient data acquisition and minimal interference from each modality, while hybridization of the multimodal method enables dynamic imaging data from multiple channel signal sources, enhanced image reconstruction with prior structural information, and improved quantitative information (25).

### Positron Emission Tomography/SPECT-CT and PET/SPECT-MRI

**FIGURE 1:**
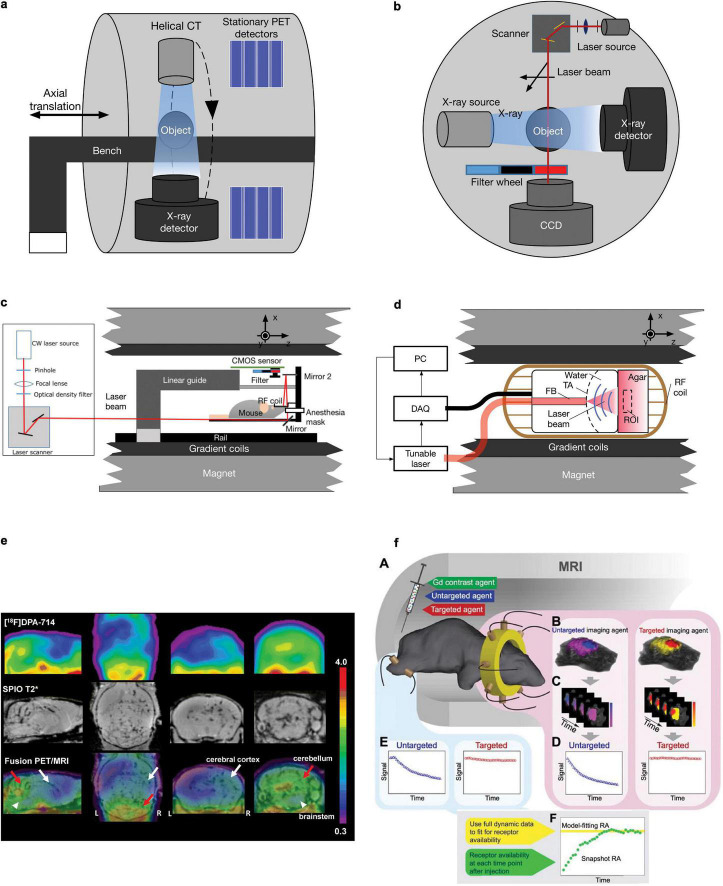
Schematics and example of different small animal hybrid imaging systems. **(a)** PET-CT uses a coaxial configuration with a helical CT scanner and stationary PET detectors aligned in parallel in one imaging chamber. A movable bench carrying the measured object allows minimal axial translation, facilitating dual-modal imaging. **(b)** A cross-sectional view of the FMT-CT configuration. In a transmission-mode FMT, the charge-coupled device detector and the illumination module, including a laser source and a scanning device, are placed on the opposite sides of the imaging object. Perpendicular to the optical path of FMT measurement, an X-ray source/detector pair is aligned in the same CT gantry. **(c)** FMT-MRI can be implemented by using an MR-compatible optical imager inserted into a preclinical MRI scanner. The major component of the insert includes a CMOS array and a customized RF coil [adapted from Ren et al. (63) with permission from Springer Nature]. **(d)** Similar to FMT-MRI, in OAT-MRI, an MR-compatible ultrasound transducer array together with the coupling medium was used as an OAT insert inside an MRI scanner. A pulsed light source and a data acquisition module (DAQ) are placed outside the MRI bore (adapted from Ren et al. (87) with permission from John Wiley & Sons, Inc. **(e)** Sagittal, coronal, and transaxial [^18^F]DPA-714 PET, SPIO T_2_*MRI, and PET/MRI fusion images of a representative experimental autoimmune encephalomyelitis mouse. PET images represent summed scans (20–50 min postinjection) normalized to the left cerebral neocortex SUV values. Increased radiotracer uptake and loss of T_2_* signal can be observed in the cerebellum (red arrow), brainstem (white arrowheads), and, to a lesser extent, right cerebral cortex (white arrow) in the experimental autoimmune encephalomyelitis mouse but not in the control. R, right; L, left. Reproduced from Coda et al. (40) with permission from Springer Nature. **(f)** MRI-FMT animal interface. (A) Illustration inside the magnet bore showing the tomographic fiber array encircling the head and a pair of optical fibers on the leg to acquire normal tissue kinetics. (B) Representative volumetric images of fluorescence activity (one frame) in the brain and tumor for both targeted and untargeted agents. Volumes such as these were acquired at approximately 0.5 Hz over the course of over 60 min, resulting in dynamic image stacks of each agent (C). Fluorescence activity was then extracted from the tumor and normal tissue to produce dynamic uptake curves, as shown in (D) and (E), respectively. Data from these curves were then used to determine RA using the model-fitting and snapshot approaches, as illustrated in (F). Reproduced from (64) with permission from Ivyspring International Publisher.

Positron emission tomography/SPECT provides quantitative *in vivo* detection of picomolar concentrations of the target within a large field-of-view (26–28). The resolution of commercially available microPET is approximately 0.5–2 mm (29–32), which is sufficient for rat brain imaging but suboptimal for mouse brain imaging considering the spillover and size of the mouse brain (10 mm × 10 mm × 15 mm) (33). The concept of combining PET/SPECT with CT was first introduced to clinical trials in the early 2000s (34). An early prototype PET/SPECT-CT scanner used a coaxial configuration and minimal axial translation of a movable couch to facilitate the sequential acquisition of PET/SPECT and CT during a single imaging session (35). The CT image serves as (1) an anatomical reference to map the molecular information given by PET and (2) an attenuation map for PET reconstruction to achieve more quantitative data (36). For hybridization of PET/SPECT and MRI, MRI provides better soft-tissue contrast without radiation and contains multiparametric (structural, molecular, and functional) readouts (25, 27, 37–39). Various MRI contrast agents, such as superparamagnetic iron oxide, can be applied to identify microglial activation/macrophage infiltration along with simultaneous PET using [^18^F]DPA-714 for glial activation in mouse models of relapsing-remitting experimental autoimmune encephalomyelitis (40) (Figures 1a,e). As a high magnetic field exists in preclinical MRI scanners, the space inside the scanner bore is significantly limited. Several PET inserts in 7T or 9.4T MRI have been reported, such as Hyperion II and MADPET4 with digital silicon photomultiplier technology (41–47). Recently, Liu et al. reported a multimodal intravital imaging system that provided a coregistered *in vivo trans-*scale and transparent platform (PET, MRI, microscopy) and quantitative evaluation of tumor anatomy, vasculature, and the microenvironment, including glucose, oxygen, and acidity metabolism (48).

### Fluorescence Molecular Tomography-CT and FMT-MRI

In addition to PET/SPECT, fluorescence imaging provides an alternative molecular imaging method with the features of high sensitivity, low cost, and non-ironizing radiation. FMT utilizes near-infrared light to penetrate living tissue up to several centimeters deep and applies a model-based reconstruction algorithm to recover the three-dimensional distribution of fluorescence probes (49). FMT-CT was first introduced by combining FMT in transmission mode with a commercial CT scanner (50) (Figure 1b); a point-shaped collimated laser source and a charge-coupled device (CCD) camera were placed on the opposite sides of the imaging object and mounted onto the CT gantry perpendicular to the X-ray instrumentation axis (51). The irregularly shaped boundary and heterogeneous inner structure can be rendered by CT and used for more accurate FMT image reconstruction (52). The FMT image quality is significantly improved with prior information from CT (53, 54). The FMT-CT hybrid system has been used for the detection of amyloid-β deposition in mouse models of Alzheimer’s disease and lung cancer (50, 55–59). FMT has been combined with MRI, such as detecting overexpressed epidermal growth factor receptors in a mouse model with gliomas (60) (Figure 1c). Similarly, an MR-compatible optical imager was inserted into a preclinical MRI scanner (61) using optical fibers or a silicon-based single-photon avalanche diode array to collect the emitted photons (61, 62). The anatomical reference obtained by MRI can be used as a prior information in a finite-element-method-based reconstruction algorithm to achieve a more accurate allocation of any fluorescent probe. In addition, dynamic image acquisition using FMT-MRI was performed to evaluate vascular perfusion and permeability in a breast tumor mouse model (63) and to quantify the target availability during therapy (64) (Figure 1f).

### Optoacoustic Tomography-CT and OAT-MRI

Optoacoustic imaging combines the rich optical image contrast and exquisite spatiotemporal resolution given by ultrasound; consequently, it has developed rapidly into a common research tool for the preclinical studies (65, 66). OAT imaging has been applied to detect molecular and functional alterations in ischemic stroke, brain tumors, and Alzheimer’s disease rodent models (22, 67–70). However, the identification of different tissues or organs is difficult due to the limited soft-tissue contrast given in OAT. Several studies have utilized hybrid imaging systems combining OAT and ultrasound that offer moderate anatomical information in small animal imaging (71–73). Although the image formation mechanisms are different in these two modalities, it is relatively straightforward to combine OAT and ultrasound, as they utilize the same transducer array and coupling medium during measurement. The development of OAT-CT and OAT-MRI is still at an early stage. Sequential-mode multimodal imaging with OAT-CT and OAT-MRI has also been reported (74–79). Coregistration of images sequentially acquired with OA and other methods is performed for volume-of-interest analysis using dedicated algorithms (80, 81), either software-based (82–85) or a hardware-assisted protocol based on stable bimodal imaging support and a rigorous data acquisition procedure (81, 86). Similar to FMT-MRI and PET-MRI hybrid systems, the combination of OAT and MRI is highly restricted by the limited space inside the MRI bore and electromagnetic interference mainly caused by radiofrequency (RF) coils. To address these challenges, the optoacoustic signal readout can be properly shielded by copper and synchronized according to different MRI sequences. More recently, a proof-of-concept hybrid OAT-MRI system that allows simultaneous recording kinetics of two contrast agents in a phantom was reported (87), for which an MR-compatible OAT insert was developed with a specifically distributed transducer array and copper-made shielding (Figure 1d). For data acquisition, the excitation laser pulse signal and MRI pulse sequence were synchronized to avoid interference between the two modalities. Hybridization with MRI greatly enhances the performance of OAT by enabling the simultaneous readings of multichannel dynamic information, including resting-state functional MRI (rs-fMRI) for blood-oxygen-level-dependent (BOLD) signals, oxygen saturation, or contrast agent biodistribution (88). Applications of OA imaging in clinical research have shown promising results and are still limited to the peripheral system (89–91). *In vivo* OA imaging in non-human primates has been demonstrated (92) and in the neonatal brain (93). A recent OA/functional MRI study of the living adult human brain demonstrated the potential of its application in the clinical neuroimaging (94, 95). However, significant challenges in skull aberrations and acoustic distortions still need to be addressed for potential clinical application and to further close the translational gap. Excitation lasers with longer wavelengths, such as in the near-infrared II window, and ultrasound transducers with lower central frequencies reduce both optical attenuation and acoustic aberration (96). The anatomical information containing both the skull and brain obtained by CT or MRI in those hybrid systems can be used for accurate modeling for light and ultrasound propagation, which can potentially improve OAT reconstruction (87).

## Translational Gaps

**FIGURE 2:**
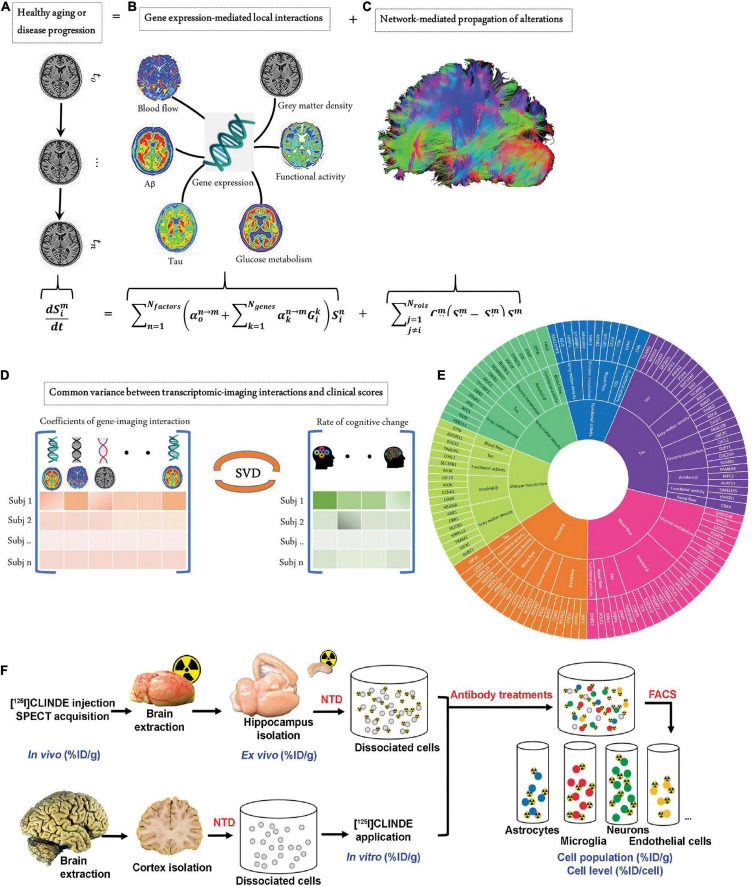
Measures to close biological gaps. **(A–E)** Integrated transcriptomic and neuroimaging data to understand biological mechanisms in aging and Alzheimer’s disease. **(A)** The longitudinal alteration of macroscopic biological factors in healthy and diseased brains due to gene-imaging interactions and the propagation of the ensuing alterations across brain networks. **(B)** Regional multifactorial interactions between six macroscopic biological factors/imaging modalities are modulated by local gene expression. **(C)** Causal multifactorial propagation network capturing the interregional spread of biological factor alterations through physical connections. **(D)** By applying a multivariate analysis through singular value decomposition (SVD), the maximum cross-correlation between age-related changes in cognitive/clinical evaluation and the magnitude of genetic modulation of imaging modalities was determined in a cohort of stable healthy subjects (for healthy aging), mild cognitive impairment (MCI) converters, and Alzheimer’s disease (AD) subjects (for AD progression). **(E)** The key causal genes driving healthy aging and AD progression are identified through their absolute contributions to the explained common variance between the gene-imaging interactions and cognitive scores. Reproduced from (148) with permission from eLife Sciences Publications, Ltd. **(F)** Overview of the fluorescence-activated cell sorting to radioligand-treated tissues (FACS–RTT) protocol. Schematic overview of the methodology used for the *in vivo*, ex vivo, *in vitro* and cellular measurement of the radioligand. % ID/cell: percentage of the injected dose/cell; % ID/g: percentage of the injected dose/g tissue weight; NTD: neural tissue dissociation. Reproduced from (120) with permission from Sage Publication.

Translational gaps exist in developing imaging biomarkers, including those (1) between *in vitro* and *in vivo* animal studies, (2) between animal and patient translation as a robust medical research tool, and (3) between research tools and integrated clinical applications (97, 98). For clinical imaging, rapid and safe processes, reliable readouts and the added value to patients (in comparison to the existing option) are important. Imaging biomarker generates added value by facilitating drug development, monitoring of treatment response (99), and reducing cost per quality-adjusted life year gained by enabling early diagnosis (100). Closing the translational gaps requires enormous technical, biological, and clinical validation and also cost effectiveness assessment (101). The lack of a satisfactory animal model is a shared problem for research on brain diseases, including stroke, neurodegenerative diseases, psychiatric diseases, and multiple sclerosis (102). Many reviews have outlined the challenges in animal models and the need for models better mimicking human diseases to improve translational power (103, 104). Using the genome engineering technology CRISPR/Cas9, humanized knock-in animal models are under rapid development. However, species differences exist in the size of the brain, anatomical structure, cerebral cortical folding, parcelation, and connectivity neuron size in humans, non-human primates, and mice (105, 106). Microglia and astrocyte in humans and mice exhibit different vulnerabilities and responses to external stressors (107, 108). The differences in gene expression and protein level between small animals and humans increase the difficulty of translation of targeting ligands (109). Moreover, there are pharmacological and behavioral differences between mice and rats, which need to be considered when interpreting the results. In addition, strain- and substrain-dependent vulnerability to pathological interventions such as permanent focal cerebral ischemia in a mouse model has been documented (110, 111). Bailey et al. showed that the C57BL/6 strain background has an influence on tauopathy progression in the rTg4510 transgenic mouse model originally of the FVB/129 background (112). Moreover, the recent findings highlighted the unique vulnerability of humans to Alzheimer’s disease or primary tauopathy, and the amyloid-β and tau deposits formed in the brains from transgenic mouse models over 1–2 years are structurally different compared with those found in aged patients (113). A similar difference has been reported for α-synuclein aggregates in patients with Parkinson’s disease (114). These differences are reflected in the divergent binding properties observed in tau/amyloid-β/α-synuclein imaging probes binding to the brains of rodent models, non-human primates, and patients (115–117). In addition to the aforementioned developments, recent single-cell tracking PET, a cellular global positioning system, was able to detect a single cell over time in a living mouse and directly related the *in vivo* imaging pattern to a single cell with its molecular profiles (118). Moreover, Tournier et al. and Nutma et al. developed fluorescence-activated cell sorting to radioligand-treated tissues (FACS-RTT) to reveal the cell origin of the radioligand translocator protein binding (119, 120) (Figure 2F).

## Awake Animal Imaging

Anesthesia leads to alterations in the brain state, cardiovascular physiology, and hemodynamic properties. Functional readouts derived from rs-fMRI (BOLD signal) and ASL-MRI (cerebral blood flow) are especially affected by awake or anesthesia status (121). In addition, PET imaging in rodents, such as [^18^F]FDG for cerebral glucose metabolism (122) and [^18^F]MPPF for serotonin 1A receptor (123), is also largely influenced by anesthesia, restraint stress, and physiological stability of the animal. A key difference between rodent and human functional imaging is the use of anesthesia to reduce motion during scanning. To overcome this gap, imaging in awake behaving rodents has been increasingly reported in the two-photon optical imaging, functional ultrasound imaging, electrophysiology, PET, and MRI in the recent years (124–127). These imaging platforms enable the integration of molecular-, behavioral-level, and circuit-level understanding of the brain. However, the duration, setup, and imaging type suitable for use in awake rodents are still limited and require extensive habituation and further development.

## Deep Learning in Multimodal Imaging Postprocessing

Deep learning-based methods have recently gained great momentum in both image reconstruction (128) and postprocessing (129, 130). Here, we focus on the DL application in image postprocessing with emphasis on image segmentation in a mono-modality and registration between different modalities. For a standalone modality such as MRI, DL has successfully been used in assisting the diagnosis of brain disease and analyzing the whole brain vasculature (131). Several pipelines for registration and segmentation of high-resolution mouse brain data onto brain atlases have been developed, such as aMAP (132, 133) and AMaSiNe (134). In addition to conventional manual feature extraction, the emerging applications of DL entail an enormous advancement in discovering new characteristic features in data (135–137). Given sufficient training, it can learn complex non-linear functions from high-dimensional and unstructured data (138). The potential of artificial intelligence has already been revealed in medical applications such as computerized diagnosis and prognosis (139). Regardless of sequential-mode multimodal imaging or truly hybrid systems, developing software-based algorithms for accurate and automatic registering molecular information with structural information is of high interest. For small animal PET, SPECT, CT, and MRI data, several established pipelines and commercial software such as ANTs, statistical parametric mapping (SPM), AFNI, PMOD, etc., have been established and routinely used (140–142). For OAT data, challenges remain in reconstruction processing, including segmentation and registration. Different detection configurations generate OAT images with different features. As OAT images have limited soft-tissue contrast, segmentation, and registration are thus difficult and highly dependent on the user experience. Human inputs were involved in the previously reported OAT-MRI registration methods to a certain extent, such as piecewise linear mapping algorithms (82). Gehrung et al. developed an integrated protocol for OAT-MRI registration by using a customized animal holder and landmark-based registration software (86). Ren et al. reported semiautomated OAT-MRI brain imaging data registration software that used active contour segmentation as the first step and an adaptive mutual information-based registration algorithm (84). Hu et al. developed a fully automated registration method for OAT-MRI brain imaging data registration empowered by DL, which consists of (1) two U-net-like neural networks to segment OAT and MRI images (71) and (2) an adaptive neural network to transform these generated OAT/MRI masks (reference). The accuracy and robustness of such DL-based registration have been shown to be comparable with classic methods but at a much higher speed without manual efforts on either landmark selection or boundary drawing (143).

## Transcriptomics

With the affordability of single-cell sequencing, metabolomics, and transcriptomics (144–146), deep phenotyping techniques allow us to elucidate the similarities and differences in the genetics (147) and protein expression in animal models and humans (109, 148–151). Single-cell transcriptomic profiling of aging and Alzheimer’s disease combined with data from amyloid and tau PET, [^18^F]FDG PET, and rs-Fmri, and structural MRI to unveil the gene and macroscopic factor interactions and the biological mechanisms underlying Alzheimer’s disease (148) (Figures 2A–E). Spatially resolved transcriptomics in particular, such as fluorescence *in situ* hybridization, showed promising application in convergent cellular, transcriptomic, and molecular neuroimaging data (149, 152, 153). Allen brain transcriptomics mouse and human datasets provide an excellent platform for exploring the link between mice and humans, integrated with connectivity and histology data (154). Other gene expression databases, such as ‘‘Brain RNA-Seq’’^1^ (109) and machine-learning models to improve mouse-to-human inference ‘‘Found In Translation,’’^2^ non-human primate platforms (155) are available to facilitate the interpretation of experimental observations. In addition, the uptake of several tracers, such as [^18^F]GE-180 (for glial activation) and [^18^F]FDG (for cerebral glucose metabolism), is influenced by sex (156–158). Further transcriptomic analysis may reveal sex-specific molecular differences associated with the uptake of different tracers.

## Organ-On-Chip Pharmacokinetics

There is a continued need for small animals as a powerful model system to advance neuroimaging and translational brain research (159). The biodistribution and pharmacokinetic information of pharmaceuticals in rodents and *ex vivo* target validation are prerequisites for phase 1 studies. Recent developments in organoids from induced pluripotent stem cells have introduced a paradigm shift for drug development (160). Cerebral organoids and blood–brain barrier organoids that mimic mouse or human physiology for investigating the permeability of compounds have been developed (161). With the goal of implementing the 3R principle (replacement, refinement, and reduction), recent efforts have been made to assess the behavior of imaging ligands using organ-on-chip systems employing organoids (162, 163). In addition, several recent studies reported systems for the characterization of cellular pharmacokinetics based on microfluidic systems, including a continuously infused microfluidic radioassay (164), microfluidics-coupled radioluminescence microscopy (165), and droplet-based single-cell radiometric assay (166). Further research on *in vitro in vivo* extrapolation will facilitate the translation and development of imaging ligands toward clinical application.

## Discussion

Imaging methods that non-invasively record rapidly changing functional and molecular imaging data have been crucial for our understanding of human and small animal brains. Recent developments in optogenetics, chemogenomics, two-photon microscopy, and all optical interrogation have improved the understanding of physiological and pathological processes in the rodent brains (167–175). However, these imaging methods are limited to preclinical applications. To further improve the translational power of PET, MRI, and CT and optical imaging, we suggest the following considerations:

### Standardization, Quality Assurance, and Quality Control

Standardization of imaging protocols and data analysis: The standardization of PET, CT, and MRI procedures in humans is more advanced than that in preclinical imaging and is a prerequisite for clinical trials. Even for structural MRI, scan session, head tilt, interscan interval, acquisition sequence, and processing stream have been found to influence the imaging results (176). The standardization of preclinical PET-CT/PET-MRI protocols, including CT, absorbed dose guidelines, has not been fully established. The updated ARRIVE guideline 2.0 provides a general checklist of *in vivo* experiments to facilitate the reproducibility of the results and methodological rigor (177, 178). Previous systematic reviews have systematically evaluated the choice of anesthetic/sedative regimen and the variation in fMRI rodent imaging (179–181). In a recent multicenter study in the United States and Europe, standardization of the preclinical PET/CT acquisition and reconstruction protocols have been shown to increase the quantitative accuracy as well as the reproducibility of imaging results (182). Similar multicenter, cross-scanner validation studies are needed for PET, SPECT, MRI, ultrasound, CT, optical imaging, hybrid imaging, and less established imaging tools, e.g., OAT or FMT. Osborne et al. proposed useful guidance for QC and scanner calibration procedures for the preclinical imaging laboratories with a balanced cost consideration (183). In the terms of data postprocessing, recent comparisons of different fMRI processing pipelines outline the importance of consensus and move beyond processing and analysis-associated variation to increase the reproducibility in human neuroimaging data (184, 185). For mouse brain imaging, recent multicenter rs-fMRI analyses have reported common functional networks in the mouse brain (186). Further comprehensive QA/QC consensuses and multicenter studies are needed for the scanner as well as for the entire pipeline, including data acquisition, reconstruction, and postprocessing to improve reproductivity and reduce variation across different studies.

### Open Data Sharing

The open sharing of neuroimaging research data is critical to promote the reproducibility of scientific findings (187, 188). Large international initiatives in human brain imaging data sharing, such as ADNI (189), OpenNeuro (190), the human connectome project (191), and OASIS (192), have greatly facilitated the advancement of the research field. In contrast, less small animal neuroimaging data sharing has been achieved thus far. There is thus a need for promoting the sharing of mouse and rat neuroimaging data that are compliant with brain imaging data structure (BIDS) following the FAIR principles (193). Increasing the sample size of animal studies and cross-study comparisons add to the reliability of the findings (194).

## Conclusion

In conclusion, developments in hybrid imaging, deep learning in data processing, awake animal imaging, and transcriptomics have greatly improved the translation of preclinical brain imaging. Further efforts on standardization, quality assurance/quality control, data sharing, and on-chip modeling for the translation of preclinical small animal brain imaging from bench to bedside need to be undertaken.

## Author Contributions

WR and RN wrote the draft. All authors contributed to the manuscript.

## Conflict of Interest

LC is employed in Shanghai Changes Tech, Ltd. The remaining authors declare that the research was conducted in the absence of any commercial or financial relationships that could be construed as a potential conflict of interest.

## Publisher’s Note

All claims expressed in this article are solely those of the authors and do not necessarily represent those of their affiliated organizations, or those of the publisher, the editors and the reviewers. Any product that may be evaluated in this article, or claim that may be made by its manufacturer, is not guaranteed or endorsed by the publisher.

## Footnotes

1
https://www.brainrnaseq.org/

2
http://www.mouse2man.org
